# Type 1 diabetes and the challenges of emotional support in crisis situations: results from a feasibility study of a multidisciplinary teleintervention

**DOI:** 10.1038/s41598-022-12227-z

**Published:** 2022-05-20

**Authors:** Janine Alessi, Alice Scalzilli Becker, Bibiana Amaral, Giovana Berger de Oliveira, Débora Wilke Franco, Carolina Padilla Knijnik, Gabriel Luiz Kobe, Ariane de Brito, Taíse Rosa de Carvalho, Guilherme Heiden Telo, Beatriz D. Schaan, Gabriela Heiden Telo

**Affiliations:** 1 Medical Science Program: Endocrinology, Universidade Federal do Rio Grande do Sul, Rua Ramiro Barcelos, 2350, prédio 12, 4° andar, Porto Alegre, RS 90035-003 Brazil; 2 Internal Medicine Department, Hospital São Lucas-Pontifícia Universidade Católica do Rio Grande do Sul, Porto Alegre, Brazil; 3 School of Medicine, Pontifícia Universidade Católica do Rio Grande do Sul, Porto Alegre, Brazil; 4 Medical and Health Sciences Program, Pontifícia Universidade Católica do Rio Grande do Sul, Porto Alegre, Brazil; 5 School of Medicine, Universidade Federal do Rio Grande do Sul, Porto Alegre, Brazil; 6 Endocrinology Division, Hospital de Clínicas de Porto Alegre, Porto Alegre, Brazil; 7 National Institute of Science and Technology for Health Technology Assessment (IATS)-CNPq, Porto Alegre, Brazil

**Keywords:** Type 1 diabetes, Quality of life, Epidemiology

## Abstract

The association between type 1 diabetes and mental health disorders could be exacerbated in a stressful environment. This study aimed to evaluate the feasibility of a teleguided intervention on emotional disorders in patients with type 1 diabetes during the COVID-19 outbreak. This study was performed during the social distancing period in the COVID-19 outbreak in Brazil. Individuals with type 1 diabetes aged ≥ 18 years were selected to receive a teleguided multidisciplinary intervention or the usual care plus an educational website access. The proposed intervention aimed addressing aspects of mental health, diabetes care and lifestyle habits during the pandemic. The feasibility outcome included the assessment of recruitment capability and adherence to the proposed intervention. Moreover, we evaluated the presence of positive screening for emotional disorders (Self Report Questionnaire 20) after a 16-week intervention, patients’ perceptions of pandemic-related changes, diabetes-related emotional distress, eating disorders, and sleep disorders. Data were analyzed with the intent‐to‐treat principle. Fifty-eight individuals (mean age, 43.8 ± 13.6 years) were included (intervention group, n = 29; control group, n = 29). At the end of the study, a total of 5 participants withdrew from the study in the intervention group compared to only 1 in the control group. Participants who dropout from the study had similar mean age, sex and income to those who remained in the study. The analysis of mental health disorders was not different between the groups at the follow up: a positive screening result was found in 48.3% and 34.5% of participants in the intervention and control groups, respectively (P = 0.29). The intervention group felt more supported in their diabetes care during the social distancing period (82.8% vs. 48.3% in the control group, P < 0.01). Our study identified a disproportionate higher number of withdrawals in the intervention group when compared to the control group. This difference may have compromised the power of the study for the proposed assessments and should be reevaluated in future studies.

Trial registration: ClinicalTrials.gov (NCT04344210). Date of registration: 14/04/2020.

## Introduction

Type 1 diabetes mellitus is a chronic disease that is increasing in both incidence and prevalence^1^. In 2019, there were approximately 1.1 million individuals under the age of 20 years with this diagnosis^2^. This reflects an increase in the annual incidence of the disease of approximately 2–3%. Brazil has the third highest incidence of type 1 diabetes, with approximately 7.3 new cases per thousand inhabitants per year^2^. The challenges of living with diabetes are reflected in different spheres of life for those who receive this diagnosis. There is often a compromise in interpersonal relationships, financial demands and emotional overload related to dependence on continuous health care^3^. These behavioral health factors associated with living with type 1 diabetes may potentially result in mental health disorders. In line with this knowledge, depression and anxiety are two- to four-times more prevalent in those living with type 1 diabetes compared to those without diabetes^4^. In Southern Brazil, about 20% of patients with type 1 diabetes are diagnosed with depression and 40% with anxiety. Those patients who had concurrent diabetes and psychiatric illnesses also had worse glycemic control^5^.

The prevalence of emotional disorders in patients with type 1 diabetes could be even more expressive in a stressful environment, such as the COVID-19 outbreak. Several measures have been taken to prevent the spread of COVID-19, including isolation of suspected cases, tracking and monitoring of contacts, and the recommendation of social distancing, especially for high-risk groups such as patients with diabetes^6–8^. The COVID-19 measures have the potential to affect the mental well-being of these patients. A previous study that was performed by our group showed that up to 94% of patients with type 1 diabetes have positive screening results for a mental health disorder during the pandemic^6^. These data highlight the need for mental health access and support for patients with type 1 diabetes during and after this outbreak.

Teleinterventions could be used as a strategy to reduce the impact of the COVID-19 outbreak on the mental health of patients with type 1 diabetes. A pilot study performed in Italy during the lockdown period found that the use of remote glucose control systems may improve glycemic control^9^. Previous studies have also shown that telehealth strategies can result in improvements in patient satisfaction with their care and quality of life^10^. Moreover, telemedicine has the potential to increase access to healthcare, which may improve diabetes management and reduce severe hypoglycemic episodes^9–11^. The use of a multidisciplinary teleintervention has been shown to be effective in reducing mental health disorders in outpatients with type 2 diabetes during periods of crisis, such as the COVID-19 outbreak^12^. However, there are no studies to date that assessed the feasibility of performing this type of intervention in type 1 diabetes patients. This study is part of a protocol that assessed the use of teleguided interventions on emotional disorders in patients with diabetes during the COVID-19 outbreak, and presents the feasibility evaluation of the intervention in patients with type 1 diabetes.

## Methods

### Study design

We performed a study to assess the feasibility of a teleintervention on mental health parameters of patients with type 1 diabetes during an outbreak. Previous databases were used to identify potential participants for the study, which refer to databases of main institutions where patients with type 1 diabetes undergo outpatient follow-up and contained information on telephone number and recent glycated hemoglobin (HbA1c) assessment. A medical record review was later performed to identify those who met the inclusion criteria for the study. Potential participants were contacted by telephone and invited to participate in the study, and an inclusion in the protocol was performed at that time to respect social distancing measures.

### Participants

Individuals with a previous diagnosis of type 1 diabetes with regular follow-up in two public care centers in Southern Brazil were selected. Patients aged ≥ 18 years and with a measurement of HbA1c between January and March 2020 were included. The exclusion criteria were patients who had a medical history of any condition that prevented their understanding of the questionnaires (such as dementia) and interaction with researchers by telephone (such as deafness). Institutionalized and hospitalized patients at the time of inclusion were also not included. Previous diagnoses of mental health disorders were not considered inclusion or exclusion criteria.

### Enrollment and study procedures

**Figure 1:**
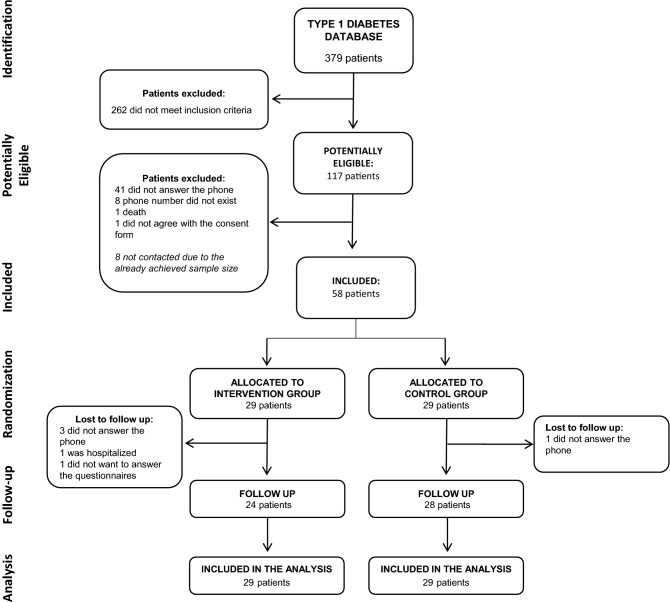
Flow diagram of the study.

Enrollment began on April 14, 2020 and ended on April 29, 2020. The first confirmed case of COVID-19 in Brazil was on February 26, 2020, and the formal recommendation for social distancing for risk groups in Southern Brazil (Porto Alegre city) started on March 22, 2020. Thus, patients were included in the study approximately 2 months after the first case of COVID-19 in the country and 1 month after the beginning of the contact restriction measures. For the identification of potential participants for enrollment, a list of all patients with type 1 diabetes mellitus who underwent follow-up at the study institutions between January 2016 and December 2019 was assessed. These participants initially identified had their electronic medical records reviewed, and those who met the inclusion criteria and had an updated telephone number were randomly contacted via telephone to invite them to participate in the study. The enrollment procedure stopped when the number predicted in the sample size calculation was officially included in the study. The details of this procedure are fully described in Fig. 1.

An inclusion questionnaire was applied when the participant was enrolled into the study. Randomization was performed for patients with type 1 diabetes in enrollment into the study, in a 1:1 ratio that was provided by the *Randomization.com* website. The electronic system generated randomization patterns for the sequence of inclusion of participants with type 1 diabetes in the study. The main researcher was responsible for generating the randomization patterns, which were performed before inclusion of participants in the study. Participants were randomly contacted, without any prior knowledge of them by the research team, and then allocated to each group based on their inclusion number and the pre-established allocation pattern. Participants who were enrolled received a second call to start the intervention procedures or to receive guidance on an educational website that was available to the control group.

### Teleintervention characteristics

A multidisciplinary team composed of 6 members (2 general practitioners, 1 endocrinologist, 1 nutritionist, 1 physical educator, and 1 psychologist) was responsible for preparing protocols for appointments that were performed remotely. The original clinical trial design envisaged a similar intervention for patients with type 1 diabetes or type 2 diabetes^12^. However, all protocols were customized for the particularities of type 1 diabetes. The objective of this strategy was to provide guidance tools and to represent a support channel for the needs of patients with type 1 diabetes during the COVID-19 outbreak. The interface protocols used are available as supplementary material.

For the maintenance of remote connections, a group of moderators, which corresponded to postgraduate students and graduate students, was responsible for mediating contact between the patients and the multidisciplinary team, as pre-planned in the initial protocol. In this study, the intervention was moderated by experienced professionals, who were attending a postgraduate course (JA, DWF, GHT, TRC). The moderators went through a training process to qualify them to make the proposed remote appointments. Then, these moderators were responsible for performing the weekly teleinterventions and discussing potential questions regarding the participants with the multidisciplinary team. An online instant messaging group was created so that the multidisciplinary team could instantly respond to the moderators' demands. The moderators were responsible for transmitting the information to participants, and there was no direct contact between the multidisciplinary team and the patients included. The participants were assigned to a specific moderator, who accompanied the same participant throughout the intervention. The assignment was performed based on the participant inclusion number, matching with a list of moderators in alphabetical order.

The duration of the proposed intervention was 16 weeks. The main pillar of this intervention was the provision of weekly telephone contacts between patients and health professionals. Each remote appointment was scheduled to last about 10 min and aimed to address different topics related to the control of diabetes, the presence of emotional overload, and the maintenance of healthy habits during the outbreak.

In addition to developing the protocols, the multidisciplinary team was also responsible for addressing diabetes care demands during the study period. Moderators could access the multidisciplinary team at any time during the follow-up period to address specific patient demands. During the remote contacts, patients were routinely asked for reports on glycemic controls, and were encouraged to maintain good adherence to treatment during each call. Prescription adjustments were discussed with an endocrinologist if recurrent hypoglycemia was reported.

Participants who were randomized to the control group received the usual care during the outbreak, in accordance with the pandemic-related restrictions. For this group, a website was made available with recommendations about maintaining healthy habits during crisis situations. This proposal aimed to offer a reliable source of information during the outbreak for these participants without interacting directly with them.

### Outcome measures

Emotional disorder outcomes and changes that occurred during the pandemic were assessed using specific questionnaires, which were applied via telephone calls. All participants were evaluated when they were enrolled into the study (baseline) and after 16 weeks of intervention (follow-up). For the evaluation of the study outcomes, telephone calls were made for the application of the questionnaires by the group of researchers of the project, and moderators were not responsible for applying the questionnaires to their respective patients. Subsequently, a physician blinded to the participants responses was responsible for evaluating the questionnaire final scores.

The study outcome was the assessment of recruitment capability and adherence to the proposed intervention (feasibility outcome). Moreover, we evaluated the presence of a positive screening for emotional disorders at the 16-week follow-up. The Brazilian version of the *Self Report Questionnaire*-20 (SRQ-20) was used for this evaluation, and a positive screening result was considered if the score was ≥ 7^13^. The choice of this questionnaire was based especially on the wide range of psychiatric disorders that it assesses (anxiety disorders, depression, and somatoform disorders) compared to other mental health scores. In addition, an assessment of differences between the groups for diabetes-related emotional distress, eating disorders, and sleep disorders was performed using screening tools. The Brazilian version of the *Problem Areas in Diabetes Scale* (B-PAID) was used to evaluate diabetes-related emotional distress (considered positive if the score was ≥ 40)^14^. The *Brazilian version of the Eating Attitudes Test* (EAT-26) was used to assess eating disorders (considered positive if the score was ≥ 20)^15^. The *Brazilian version of the Mini Sleep Questionnaire* (MSQ) was used to evaluate sleep disorders screening (considered positive if the score was ≥ 31)^16^.

Finally, an evaluation of patients’ perceptions (subjective assessment of changes that occurred with the pandemic in relation to eating habits, physical activity, glycemic control and mental health) was performed as pre-planned in the protocol. For this assessment, participants were asked to give a score (0 to 10) for adherence to diet, maintenance of physical activity, glycemic control, and mental health according to their impression before and during the pandemic (follow-up period). Moreover, psychosocial aspects and perceptions about diabetes care during the pandemic were assessed by asking the participants about the presence of respiratory symptoms. Finally, social distancing measures, financial and medical assistance difficulties that may have occurred during the outbreak period were asked with yes/no answer options.

### Demographics and clinical data

Personal information, such as age, marital status, race/ethnicity, diabetes duration, disease complications, current medications, and psychiatric history were obtained from each patient’s medical records and then verified by the participant. The HbA1c (high-performance liquid chromatography method) result was obtained from records and collected between January and March 2020. Diabetes complications were evaluated using the presence of retinopathy, which was considered based on the last fundus examination. For neuropathy, the presence of a previous diagnosis or an altered monofilament 10-g test result at a medical appointment was considered. For diabetic kidney disease, the presence of macro/microalbuminuria or glomerular filtration rate lower than 60 ml/h registered in medical records were considered. A history of coronary heart disease, stroke, heart failure, or peripheral arterial disease that was recorded in the patients’ medical records indicated cardiovascular disease.

### Power estimations

This study was designed to assess the feasibility of the proposed teleintervention, which was designed to improve mental health parameters in patients with type 1 diabetes. We performed the sample size calculation for two-sample design according to the superiority by a margin test for difference between two proportions described by Chow et al.^18^. A previous study found that, with the use of a remote intervention in patients with type 1 diabetes, changes were significantly greater in the intervention group compared to a control group. Assuming that 28% of the subjects in the control group and 64% in the intervention group improved mental health parameters with a remote teleintervention^17^, and after applying continuity correction, the study would require a sample size of 45 individuals for each group to achieve a power of 80% and a level of significance of 5% for declaring that the intervention is superior to the control group at a 10% margin of superiority.

### Statistical analysis

We used SPSS v.22 software (IBM Corp., Armonk, NY, USA) software for the analyses. Participants’ characteristics data were reported as the mean ± standard deviation (SD) if the data were normally distributed. Differences between groups for baseline data were evaluated using an unpaired *t* test and the Mann–Whitney *U* test for continuous variables and the Chi-square test was used for categorical variables.

Data related to adherence and feasibility of the proposed intervention are presented descriptively. Furthermore, secondary data were analyzed using the intention‐to‐treat principle. We used the Markov Chain Monte Carlo multiple imputation algorithm to deal with the missing data. Clinical and psychosocial aspects and perceptions about diabetes care during the study were assessed using the Chi-square test. Data on patients’ perceptions of changes in habits that occurred during the pandemic were reported as the median ± interquartile range (IQR), and analyses were performed using the Mann–Whitney *U* test for the between-groups comparisons and the Wilcoxon Rank test for the within-group comparisons. Results of the questionnaires were analyzed for the presence of a positive screening result for the disorder based on previously cited cutoff values. Comparisons of positive screening between groups were performed using the Chi-square test/Fisher’s exact test and comparisons of within-group data were performed using the McNemar’s test. Comparisons within groups were performed *post hoc* and sought to assess changes from baseline to follow-up within each arm of the study. Two-tailed tests were used to determine significance at the 5% level.

### Ethics approval and consent to participate

The informed consent form was read by the telephone contact for all participants who were included in the study. The terms were read and applied by the postgraduate students of the research group. Agreement was registered using an audio recording or an electronic message. All methods were performed in accordance with the relevant guidelines and regulations, and was approved by the institutional ethics committee (Comissão Nacional de Ética em Pesquisa—Nº 4059760). This trial was registered at ClinicalTrials.gov (registration: NCT04344210). This reporting follows the CONSORT statement^18^.

### Consent for publication

All authors have reviewed the final version of the manuscript and agreed with the publication of the results presented.

## Results

Overall, 117 potentially eligible patients were identified, and the enrollment stopped when 58 individuals with type 1 diabetes provided informed consent. Overall, participants had a mean age of 43.8 ± 13.6 years, 50.0% were female, and 31.0% were married. Most participants were white and had a low-to-middle income. The mean diabetes duration was 25.2 ± 11.6 years and the HbA1c value was 8.7 ± 1.5% (72.0 ± 16.4 mmol/mol). A previous depression diagnosis was found in 25.9% and a previous anxiety diagnosis in 3.4% of participants. There were no differences between groups regarding the baseline characteristics (Table 1).

**Table 1:** Baseline characteristics of study participants.

	Total (n = 58)	Control (n = 29)	Intervention (n = 29)	P value
Age (years)	43.8 ± 13.6	43.9 ± 14.0	43.8 ± 13.4	0.99
Sex (% female)	50.0%	55.2%	44.8%	0.43
Race/ethnicity (% white)	96.6%	96.6%	96.6%	1.00
Marital status (% married)	31.0%	37.9%	24.1%	0.26
Low-middle income*	79.3%	82.8%	75.9%	0.52
Regular work	63.8%	65.5%	62.1%	0.79
Diabetes duration (years)	25.2 ± 11.6	24.5 ± 12.2	26.0 ± 11.0	0.61
HbA1c pré-pandemic (%) (mmol/mol)	8.7 ± 1.5 72.0 ± 16.4	8.9 ± 1.4 74.0 ± 15.3	8.5 ± 1.5 69.0 ± 16.4	0.28
HbA1c follow-up (%)^#^ (mmol/mol)	8.2 ± 2.3 66.0 ± 25.1	8.5 ± 2.3 69.0 ± 25.1	7.9 ± 2.2 63.0 ± 24.0	0.32
**Diabetes complications**				
Retinopathy	50.0%	44.8%	55.2%	0.43
Neuropathy	25.9%	24.1%	27.6%	0.76
Diabetic renal disease	36.2%	34.5%	37.9%	0.79
Weight pré pandemic (kg)	69.3 ± 16.9	67.1 ± 20.5	71.6 ± 11.8	0.33
Weight follow up^#^ (kg)	69.7 ± 17.6	69.4 ± 15.8	69.9 ± 19.9	0.91
Systemic arterial hypertension	43.1%	51.7%	34.5%	0.19
Cardiovascular disease	12.1%	13.8%	10.3%	0.69
ACE or ARB inhibitors use	31.0%	37.9%	24.1%	0.26
Statins use	39.7%	37.9%	41.4%	0.79
ASA use	15.5%	17.2%	13.8%	0.72
Depression	25.9%	34.5%	17.2%	0.13
Anxiety	3.4%	3.4%	3.4%	1.00
Other psychiatric condition	8.6%	6.9%	10.3%	0.64
Antidepressant use	5.7%	3.6%	8.0%	0.49

Data are mean ± standard deviation or %. α ≤ 0.05 indicates significant difference. *HbA1c* glycated hemoglobin, *ACE* angiotensin-converting enzyme, *ARB* angiotensin II receptor blocker, *ASA* acetylsalicylic acid. ^#^Results for 49 participants, who attended the face-to-face assessment for laboratory tests and weighing between 2 and 5 months after the study.

### Study outcomes

#### Feasibility evaluation

Sixteen remote appointments were planned, but the median number of calls that were received by the participants in the intervention group was 13.0 (IRQ 11.3–15.8). The moderators made three contact attempts weekly if the participant did not answer on the first call. Only three participants received fewer than ten calls due to the difficulty of contacting them (one participant received nine calls, one participant received eight calls, and one participant received only two calls). Four participants needed clinical support to adjust their insulin dose due to recurrent hypoglycemia, which was discussed with an endocrinologist from the multidisciplinary team. At the end of the follow-up, six participants withdrew from the study: four participants did not answer the phone (three in the intervention group and one in the control group); one participant was hospitalized; and one participant did not respond to the final questionnaires and requested to be removed from the study (the latter two participants were both in the intervention group) (Fig. 1). Participants who withdrew from the study had similar mean age, sex, family income, and baseline depression screening scores to those who remained in the study.

#### Emotional disorders evaluation

**Figure 2:**
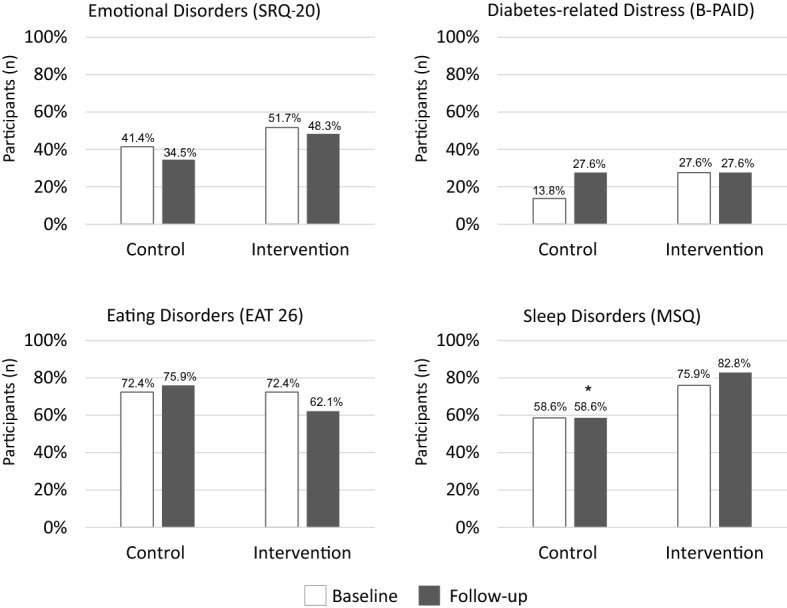
Participants with positive screening for proposed assessments, based on cutoff values and comparison between intervention and control groups. Legend: Number of participants who present positive screening based on pre-established cutoff values. For the evaluation of emotional disorders, a score greater than or equal to 7 on SRQ-20 is considered positive. Diabetes-related emotional distress is considered when the B-PAID score is greater than or equal to 40. The presence of positive screening for eating disorders is considered when the EAT 26 score is greater than or equal to 20. A positive screening for sleep disorders is considered when a score greater than or equal to 31 is present in the MSQ. *P = 0.04 for comparison between groups after the intervention.

For emotional disorders (SRQ-20 questionnaire), a positive screening result was found in 51.7% and 41.4% of participants in the intervention and the control group at the baseline, respectively (P = 0.43). In the follow-up, a positive screening result was found in 48.3% and 34.5% of participants in the intervention and control groups, respectively (P = 0.29) (see Fig. 2). For within-group analyses, there was no difference in the baseline and follow-up results (P > 0.99) for within-group comparison in the intervention group and P = 0.79 for within-group comparison in the control group.

#### Psychosocial aspects and perceptions about diabetes care during the pandemic

During the pandemic, there were 29.3% of participants who followed the guidance of complete social distancing and 58.7% who followed the guidance only partially (maintained basic activities). Around 70.7% had contact only with the family during the study period. For diabetes care, 38.0% received remote care from their attending physician and 22.4% considered their medical care to be worse during the outbreak. Additionally, 20.7% of the participants reported difficulties obtaining medical assistance during the period, and 19.0% reported difficulties in getting medication prescriptions. Most participants (53.5%) reported financial difficulties and 6.9% lost their jobs during the pandemic. The two groups were comparable in most of the evaluated characteristics. However, participants from the intervention group reported more frequently that they felt supported in their diabetes care during the social distancing period (82.8% vs. 48.3%, P < 0.01), (see Table 2).

**Table 2:** Assessment of clinical, psychosocial aspects and perceptions about diabetes care after 16 weeks of follow-up during the COVID-19 outbreak.

	Total (%)	Control (n = 29) (%)	Intervention (n = 29) (%)	P value
**Followed social distancing**^**$**^				
Partially	58.7	62.1	55.2	0.48
Totally	29.3	31.0	27.6	
None	12.1	6.9	17.2	
**Maintained social contact**				
Only family	70.7	72.4	69.0	0.22
Family and friends	13.8	6.9	20.7	
None	15.5	20.7	10.3	
Had respiratory symptoms	29.3	31.0	27.6	0.77
Had COVID-19 infection confirmed	3.4	3.4	3.4	> 0.99
Was hospitalized	1.7	0.0	3.4	0.31
Felt supported about the diabetes care	65.6	48.3	82.8	< 0.01
Received remote care from the attending physician	38.0	34.5	41.4	0.59
Considered medical care worst during the outbreak	22.4	31.0	13.8	0.22
Had difficulties getting medical care	20.7	27.6	13.8	0.20
Had difficulties getting medication prescriptions	19.0	20.7	17.2	0.74
Became unemployed during the outbreak	6.9	6.9	6.9	> 0.99
Had financial difficulties	53.5	44.8	62.1	0.18

Data are %. α ≤ 0.05 indicates significant difference. ^$^Partial social distancing includes patients who left home only for basic activities, such as market, pharmacy and health care. Total social distancing includes patients who followed the orientation of home-staying only.

#### Changes in habits during the COVID-19 outbreak

Participants were asked to provide a score, from zero to ten, for the quality of some aspects in their daily routine before and during the COVID-19 outbreak. Comparisons between the groups in relation to the evaluation periods showed a similarity in self-reported scores for eating habits, physical activity, glycemic control, and mental health. For within-group comparisons, both control and intervention groups reported worsening of physical activity and mental health parameters during the pandemic. For the self-reported score for physical activity, the intervention group had a median change score of − 1.0 (IQR − 2.0 to − 1.4), P for within-group comparison = 0.02, while the control group had a median change of − 2.0 (IQR − 1.0 to − 2.0), P = 0.001. For mental health, the intervention group had a median change score of − 1.0 (IQR − 1.0 to − 1.0), P = 0.02, while the control group had a median change score of − 2.0 (IQR − 2.0 to 0.0), P = 0.01.

#### Diabetes-related emotional distress

For diabetes-related emotional distress between groups (B-PAID questionnaire), the presence of a positive screening result was found in 27.6% and 13.8% of participants in the intervention and control groups at the baseline, respectively (P = 0.20). In the follow-up, a positive screening result was found in 27.6% and 27.6% of participants in the intervention and control groups (P > 0.99), (see Fig. 2). There was no difference in the within-group analyses for the baseline and follow-up results.

#### Eating disorders

When assessing eating disorders between groups (EAT-26 questionnaire), the presence of a positive screening result was equal (72.4%) in the intervention and control groups (P > 0.99) at the baseline. At the follow-up visit, a positive screening result was found in 62.1% and 75.9% of participants in the intervention and control groups, respectively (P = 0.26) (see Fig. 2). The within-group analyses showed that there were no changes in screening for the intervention and control groups between the baseline and follow-up responses.

#### Sleep disorders

When evaluating sleep disorders between groups (MSQ questionnaire), a positive screening result was found in 75.9% and 58.6% of participants in the intervention and control groups at the baseline, respectively (*P* = 0.16). At the follow-up visit, a positive screening was found in 82.8% and 58.6% of participants in the intervention and control groups (P = 0.04) (see Fig. 2). When using a multivariable model with the inclusion score, there was no difference between groups OR 3.43 (95% IC, 0.9–11.8). There was no change in the within-group difference at baseline and follow-up in the intervention and control groups (P = 0.73 and P > 0.99, respectively).

## Discussion

The psychological impact of the COVID-19 pandemic has the potential to generate lasting and persistent damage to the population. This study assessed the feasibility of a telehealth intervention on emotional disorders in patients with type 1 diabetes during the social distancing period. Our study identified a disproportionate higher number of withdrawals in the intervention group when compared to the control group. This difference may have compromised the power of the study for the proposed assessments. When trying to understand possible baseline characteristics that may have mediated these dropouts, no significant differences were found comparing to those who remained in the study. Thus, we hypothesized that there is possibly less acceptability for this type of teleintervention among patients with type 1 diabetes.

Remote strategies are aids in the care of type 1 diabetes. Different studies have shown a reduction in episodes of hypoglycemia and an improvement in the quality of life related to this type of intervention^9–11^. However, improving mental health parameters in these patients is still a challenge. In our study, we found no significant differences in screening for mental health disorders with the teleintervention tested. We believe that this may have occurred due to the greater number of dropouts in the intervention group, which may have compromised the evaluation performed. Interestingly, a similar intervention was developed for patients with type 2 diabetes, and it was effective in reducing the prevalence of mental disorders by up to 36% during the pandemic^12^.

The main studies on teleinterventions that showed positive results in mental health issues included patients with type 1 diabetes or type 2 diabetes^17,19–22^. It is possible that the positive results in these studies could be mediated mainly by patients with type 2 diabetes who seem to respond more positively to the teleinterventions. Some factors could explain this difference between the types of diabetes. First, it is possible that, because these individuals have lived with type 1 diabetes for a long time, they have a greater capacity for self-care, autonomy, and security in relation to their diabetes care^23^. Thus, providing lifestyle and diabetes care strategies remotely may be insufficient to mitigate the effects of the outbreak on these patients’ mental health. Second, it is possible that these patients, who are already emotionally fragile, need a longer intervention time to show significant emotional benefits. Third, it is possible that the younger age of patients with type 1 diabetes makes them more psychologically resilient to the emotional impact of an outbreak^24^. In this case, the prevalence of mental health disorders could reflect an already chronic condition, requiring more complex strategies to mitigate its effects. In addition, it is possible that patients with type 1 diabetes do not perceive themselves as part of the group that is at a higher risk for the disease, and thus, they are affected less by this situation. Even so, the differences in the response to teleintervention use between patients with type 1 or type 2 diabetes is thought-provoking and deserves to be better understood.

Support for diabetes care during crisis situations can directly impact glycemic control in patients with type 1 diabetes. Previous studies showed that patients who receive support and maintain regular contact with health professionals through telemedicine have an improvement of up to 0.91% in HbA1c levels^25,26^. Other studies show that this impact can also be reflected in other areas including control of blood pressure and weight as well as dyslipidemia, and it also promotes a better quality of life^27,28^. In our study, patients who maintained regular contact with health professionals reported that they felt almost twice as much support in their diabetes care during the period of social distancing, despite a significant number of withdrawals having occurred in the intervention group. The impact that this contact will have in terms of glycemic control in this context remains hypothetical, and studies evaluating metabolic outcomes are necessary to better understand this effect.

Studies that were performed during the COVID-19 pandemic showed that over 20% of patients with pre-existing psychiatric disorders reported worsening of their symptoms while social distancing^29^. In our study, over 40% of the participants had a positive screening result for emotional disorders after 20 weeks of social isolation in Brazil, reaching almost 70% of eating and sleep disorders. There was a tendency toward worsening sleep parameters in the intervention group at the end of the follow-up. However, this result seems to be mediated by a difference that already existed at baseline; although not statistically significant, the absolute number was already large. When the difference in the same group was evaluated by comparing the baseline and follow-up results, there were no significant changes in the prevalence that was present in both within-group analyses, which reinforces the impression that the difference that was found is due to chance or mediated by a small difference that already existed at baseline. Thus, the high prevalence of these disorders is an alert for health professionals, who must be attentive to signs of intense suffering and provide specialized mental health care.

This study has some limitations. Although the number of participants was in accordance with the calculated sample size, we considered that a relatively small sample was included in this study. Considering the number of dropouts in the follow-up that occurred especially in the intervention group, and less frequently in the control group, it is possible that the power for the assessment of the additional outcomes may have been compromised. In addition, the scales that were used to assess emotional disorders were designed and validated for self-application. Because the questionnaires were applied remotely to preserve patient safety during the pandemic, and mediated by a third individual, it may result in difficulty for patients to be completely honest in their responses, and it could be a potential source of bias. The scales that were used to assess psychiatric disorders are screening tools and have no diagnostic value. Finally, the inclusion of a sample from only two university hospitals in the same region of Brazil may also compromise the external validity of the study.

The COVID-19 outbreak has the potential to generate negative mental health outcomes for patients with type 1 diabetes. We evaluated the feasibility of a teleintervention study in reducing the impact of the pandemic on the mental health of these patients. Among the challenges experienced, there was a higher proportion of dropouts among participants in the intervention group compared to the control group, which are not justified by demographic and clinical differences between the participants. These unilateral dropouts may have compromised the study's power to assess the effect of the proposed teleintervention, which should be reevaluated in future studies. Even so, our results point to a possible positive effect in making the teleintervention group felt more supported in their diabetes care during the pandemic and should encourage future strategies studies to support patients with type 1 diabetes in crisis situations.

## Supplementary Information

Supplementary Information.

## Data Availability

Deidentified data are available upon justified request to the e-mail address of the main researcher and with a signed data access agreement.
