# Supplementary material for: Type 1 diabetes and the challenges of emotional support in crisis situations: results from a feasibility study of a multidisciplinary teleintervention
Source: Sci Rep. 2022 May 20;12:8526. doi: 10.1038/s41598-022-12227-z (PMC9120802; doi:10.1038/s41598-022-12227-z)
Supplement: Supplementary file 1 — Supplementary Information. [file 41598_2022_12227_MOESM1_ESM.docx]

**INTERVENTION PROTOCOLS**

**INTERVENTION WEEK 1**

Good afternoon Mr_______. This is ________, from the research on diabetes and coronavirus. How are you? I am contacting you to let you know that you were drawn to receive calls during the quarantine period. In these calls we will try to help you in several questions. Can you speak now?

Today I wanted to talk a little bit about your diabetes. In order for me to try to help you, I need to understand a little better how your diabetes care is going, especially during this period of isolation. How about, to begin with, do you tell me a little bit about what has been the hardest part about your diabetes? You can give me examples, if it gets easier.

(ANSWER to "FOOD"): In fact it has been difficult to go to the market as often as before, and by staying at home for longer it is quite possible that you will end up giving in to some less nutritious foods. There are some things that can help to improve this. One idea would be to make a shopping list so you don't forget anything and don't have to be back on the market many times. You can even ask for help from a family member or neighbor to go to the market when you need it. In this list, you can place healthier foods, such as whole products, fruits, vegetables, always trying to avoid processed foods. If you get used to eating healthier foods at home, it will make a big difference for diabetes control.)

(ANSWER to "PHYSICAL EXERCISES"): It has been difficult to maintain the exercise routine at this time. But there are several options for activities that you can do even indoors: jumping rope, dancing, climbing stairs, doing sit-ups or stretching are some options. We have a list of exercises that can be done at home. I can send it to you by email, if you are interested. I'm sure you will find our tips very useful for you to stay active right now. What do you think?)

(ANSWER to "GET MEDICATIONS"): What is the main difficulty you have had in this regard? Is it getting to the pharmacy to pick it up or is it because you no longer have a prescription? If the problem is to get the medications, is there someone in your house who can help? Or a neighbor with whom you have more proximity to whom you can ask? I think it's worth trying to ask for help right now. If the problem is the prescription, we can help. Can you send us a photo of the recipes by email so that we renew for you?

(ANSWER to "LACK OF MEDICAL CONSULTATIONS": It has been difficult to get consultations at this time, but this is for the safety of all of you. What would you expect from the medical consultation at this time? Can we, despite the limited distance, take your questions and help at least a little in this part?)

(ANSWER to "FEAR, ANXIETY, CONCERN"): It has been a very difficult time for all of us, Mr______. Some things can help, at least a little bit, in that part. Have you kept in touch with your family and friends? This is something that usually helps a lot, even if it is just making phone calls from time to time. Another thing is that you don't stop doing the things you did before, always trying to adapt so you can do it without leaving the house. Try to keep yourself busy, play some card game, do crochet, whatever you like. Try to do new things to get out of the routine, maybe learn a different game or learn to cook a new recipe ... Use your creativity to try to keep your mind busy. And, if you like to watch TV, try to avoid listening to news about the coronavirus all the time, this may end up making you even more worried.)

(ANSWER TO "CONTROL GLUCOSE"): We face many challenges to control glucose during quarantine. There are several things that can be done to improve this part. Do you usually measure blood sugar with the monitor? If so, taking measurements and writing down at least 4x a day in a notebook can help you know how your glucose is doing and how we can improve it. In addition, maintaining a balanced diet according to what your doctor recommends is very important. As lately it has been difficult to go to the market often, a suggestion is that you make a shopping list so that you don't forget anything and can go to the market a few times during quarantine. In this list, you can include healthier foods, such as whole products, fruits, vegetables, always trying to avoid processed foods. If you get used to buying healthier foods at home, it will make a big difference in controlling your diabetes. Another very important thing is that you stay active, even without leaving home. There are several exercises that can be done indoors, such as stretching, sit-ups, dancing, climbing stairs. If you want, I can email you a list of suggestions for things you can do at home, what do you think?

(ANSWER "REMEMBER TO TAKE MEDICATIONS"): I understand that it seems difficult to remember to use all medications at the right time, but it is very important that we try to improve this to better manage your diabetes. Forgetting to take your medicines or taking them at the wrong times can cause your blood sugar to reach levels that are dangerous for you. How about, to help you remember, you put an alarm clock on your cell phone for the time to take your medication? If you are unable to do this, you can ask someone who lives in the same house to help with that part. Or you can set up a table with all the medicines you need to use at home and get used to being with her always around. During the quarantine, that you will be more at home, it is a great time for us to start doing this and improve this part. What do you think?

Is there anything else that you think you could do differently to improve your diabetes control? There must be at least 1 or 2 things that go through your head ...

Do you feel that changing these things to improve diabetes is important?

What is keeping you from achieving these things so far?

Mr______, we are also very concerned about all these things that you have told us. I know that it is very difficult to get medical appointments during the quarantine. But the good news is that much of the solution to control diabetes depends only on you, without even leaving your home. If we can work together on a strategy to improve these things that are being difficult, I am sure that you will be able to do this, even on your own. But I need you to be motivated, just as I am to help you! How do you think you can start to change that? What do you think you can do TODAY so that it starts to improve little by little? (insist until he answers something)

Very well, I think you are on the right path. So, the next time I call, I want you to tell me if this strategy is working and if you are managing to put it into practice in our agreement, okay?

Before I finish, let me ask just two more things (ONLY IF THE PATIENT DID NOT MENTION THAT PREVIOUSLY). Do you usually use insulin to treat your diabetes? (IF ANSWERED NO - skip question)

(ANSWERED YES): Do you have the device for measuring blood sugar at home? Do you usually check your blood sugar with the device? For us to know how your diabetes is doing, it would be important that you measure at least once in a while. If you have the device at home, who knows, you may write down how much you are giving at least before having breakfast about 3 times a week and when I call you again next week, you will tell me how it turned out, what do you think?

Have you been able to get your medications at the clinic or pharmacy? Are you having any difficulties with this? Are your prescriptions still valid? (If they say they have no prescriptions, remember that the prescriptions are valid for 1 year during the pandemic. If they still say that the pharmacy is no longer releasing them, let them know that we can provide a prescription by email, just request that they send a picture of the previous prescription by email to janinealessi@gmail.com to receive the new prescription and explain that this will only be possible during quarantine).

Is there anything else you would like to ask me or could I help you with at that moment?

So, Mr______. Before hanging up, I would like you to summarize for me: what is the message that you are going to take from our conversation today? Very well. Next week, you tell me if it worked. That's it for today. Thank you very much for your attention, I hope our conversation has helped in some way. If you have any difficulties during the week, you can write it down on a paper and ask me on the next call, okay? Have a great day.

**INTERVENTION WEEK 2**

Good afternoon Mr___, how are you? (Briefly resume the previous call). First I would like to know about our agreement from last consultation: did you manage to do ____ (remind him what you agreed)?

(Positive reinforcement = congratulate him if he did it, encouraging him to keep the changes. If he didn't, remind him that he may still be able to do it in the next few days, that it only depends on him and that we trust his potential.)

(Present the purpose of the call): The purpose of our call today is to talk a little bit about feelings of anxiety and concern that you may be experiencing at this time, especially due to the isolation and the coronavirus. Would you like to hear some suggestions that we have to improve this part or do you think that this is not a problem and prefer to focus on other things? (If he answered that it is not necessary, do not force it. Ask about how the diabetes is going, about the treatment goals and the difficulties he might be having. Something very general and similar to what was the first week. - If he answered yes, follow below.)

(Prepare notes) In our conversation today I will say several important things that you could write down, so that you don't forget. Do you have any vision or writing problems? How about you get a pen and paper so you can write down my tips? I'll wait …

(Active listening) Very well. Tell me briefly how you are feeling about this coronavirus issue and the fact that you have to stay at home more. Have you been feeling a lot of fear or worry? Have you been able to sleep well at night? (let the patient speak for a few minutes, try not to interrupt initially).

I imagine how you are feeling, this change in routine has not been easy. And we still have to deal with a very dangerous infection, it is natural that you feel that this is taking your sleep away, making you very worried. But we are going to teach you some strategies to try to improve this during the coronavirus pandemic.

(Strategy 1 - Managing the environment): Do you usually watch news on television or other media? The truth is that the news is today the main enemy of our “mental tranquility”. The news keeps reminding our brain of the “threat” that we are living with the coronavirus. This activates a series of responses in our body that make us feel anxious. The first step in changing this is to CONTROL OUR NEWS CONSUMPTION. Some things are fundamental in this part and you should write them down. First, let's agree that you will watch news for a maximum of 2 hours a day (from morning to night). Second, let's agree that you will not watch news for at least 2 hours before going to sleep (that is, if you usually sleep at 10 pm, ideally you should stop watching news after 8 pm) - this will prevent negative thoughts in your head at bedtime, causing discomfort and disrupting sleep. Can you do that?

(Strategy 2 - Distraction) Reducing news consumption is very important, but that alone will not be enough. Can we agree that, thirdly, whenever thoughts of concern and fear about the coronavirus arise in your head, you will automatically shift your focus from thinking to something good? Every time you start to get anxious, you should close your eyes, take a deep breath, and make a mental list of 3 things you like most, that make you most happy. What do you think?

(Strategy 3 - Social relations): What will help you most is keeping in touch with your friends. I know it is very difficult now, in the quarantine season, but talking to someone you like every day will help to alleviate this anguish that you may be feeling. Can we agree that you will try to talk to at least one person every day (it can be over the phone, or by text message on your cell phone or computer)? It has to be a person that you don't see daily. What do you think about that?

Very well. If you feel that these things have not helped and the anxiety is so great that it is making you suffer, you can always call 188. Some professionals will be available to talk to you at any time. You can also always talk to me and tell me these things when I call and I will try to help in whatever I can. Alright? Did you write down the number? And did you write down the 4 things we agreed on today? If you didn't write it down, let's remember and write it down: 1) Watch a maximum of 2 hours of news per day 2) Don't watch news 2 hours before going to sleep 3) Every time thoughts of worry and anxiety arise, close your eyes and think about 3 things that you like to do it 4) Call or send a message to a distant friend every day. Next week I will call you again and you tell me if it worked out, okay? Is there anything else you want to ask me before hanging up?

**INTERVENTION WEEK 3**

Good afternoon Mr___, how are you? Today our call will be shorter! Our goal today is to talk a little about maintaining healthy eating habits during the coronavirus pandemic. We know that it can be very difficult to maintain a varied and quality diet due to the difficulty of leaving the house that we are facing. Can you tell me how it has been for you?

Indeed, this change in routine has not been easy. But it is very important that we try to maintain a healthy diet, especially for those who have to deal with diabetes! I have some tips that may be important about this. Can I talk a little about them or do you think it is not necessary? (If he doesn't think it's necessary, don't insist. If so, move on).

I'll start with some tips, you can write them down if you want! FIRST TIP: Eat fresh, natural foods every day. Try to maintain a regular consumption of fruits, vegetables and whole grain products daily. The recommended thing is to try to eat 4 servings of fruits per day, 5 servings of vegetables, 1 to 2 servings of grains and meat at least 2 to 3 times a week. In addition to being eaten fresh, fruits and vegetables can be frozen. Another idea is to use vegetables to cook large quantities of soups and other dishes and then freeze the ready-made meal, only reheating when you eat it. This will make it last longer and provide meal options for a few days.

SECOND TIP: When it is difficult to get natural products, opt for healthy dry or canned alternatives. Canned beans and chickpeas, which provide various nutrients, can be stored for months and can be included in meals in a variety of ways. Canned oily fish, such as sardines, mackerel and salmon, are rich in protein, omega 3 fatty acids and a variety of vitamins and minerals. Dry products such as beans, legumes and grains such as lentils, peas, rice, or quinoa are also nutritious, long-lasting and tasty options.

THIRD TIP: Create a stock of healthy snacks. Instead of keeping sweets or snacks at home, choose to keep a stock of healthier options, such as nuts, yogurt (without sugar), chopped or dried fruits or hard-boiled eggs. These foods are nutritious and will keep you full for longer. Try to avoid sugary drinks and try to drink lots of water throughout the day.

These are the 3 tips I have for you today. If you want to know more about this, I can send you a list by email or by phone. What do you think? Is there anything else I can do to help you today?

**INTERVENTION WEEK 4**

Good afternoon Mr___, how are you? Today we are going to talk a little about the practice of physical exercises during this quarantine period. We know that it can be difficult to maintain regular practice during this period. To start with, how about you tell me a little about how this has been for you?

Everything that we have been experiencing lately has not been easy. But it is very important that you try to maintain a physical exercise routine, as the exercises help to control sugar levels and are an excellent tool to fight anxiety and improve moods, which is essential to face these difficult and uncertain times . I have some tips that can help you. Can I talk a little about them or do you think it is not necessary? (If you don't think it's necessary, don't insist. If so, go ahead).

I'll start with some suggestions, you can write them down if you want! FIRST TIP: have a place available at your home to perform your exercise practice at any time. Often the lack of a place available prevents us from practicing because we have to move furniture around and create an environment that allows us to move around the house every time we decide to exercise. The space doesn't have to be big, just enough for you to move around without hitting furniture. If you do not have space at home, you can perform exercises such as walking outdoors, but choose to go out at times when the circulation of people is not much, and try to keep a distance of two meters from people. Preferably, use a mask.

SECOND TIP: Try to create a routine for your body to get used to the fact that every day at that time you will be exercising. If you are having trouble keeping a schedule, or starting the practice, try doing it in a more timely manner, without having to stop doing what you are doing to go and practice. For example: practice in the intervals of a program you are watching, or practice while watching a series or movie, so it can be easier to maintain a routine.

THIRD TIP: If you haven't started exercising yet and find it a little difficult, start like this: in the first week, do 5 minutes of exercise every day. In the second week, increase to 10min. The following week, 15 min, and so on, until you reach at least 30 min of daily practice. It is important to remember that you can perform aerobic exercises, such as walking, running, skipping rope, or anaerobic exercises, such as those for weight training.

A VERY IMPORTANT detail is that you should always check your blood glucose before starting activity and, if possible, intensify monitoring during the day, especially if you have done an unusual exercise. It is also important to keep some fast-acting carbohydrates nearby to correct any hypoglycemia. And don't forget about hydration, also an important attitude to preserve immunity. These are the tips I have for you today. If you want, I can send you a list of exercises by email (IF THE PATIENT HAS NOT RECEIVED THE LIST PREVIOUSLY). What do you think? Is there anything else I can do to help you today?

**INTERVENTION WEEK 5**

Good afternoon Mr___, how are you? How was the last week? Were you able to put into practice our tips for exercising at home?

The purpose of our call today is to talk a little bit about feelings of depression, such as sadness, that you may be experiencing right now, especially due to isolation and the coronavirus. Would you like to hear some suggestions that can improve this part or do you think this is not a problem and prefer to focus on other things?

If you answered yes, proceed: Very well. Tell me briefly how and how often you have been sad or upset. Have you been unmotivated or unhappy?

I imagine how you are feeling, and usually when we feel that way, we stop doing things because everything seems impossible or too much work. Sometimes we can't even begin or try to do anything to make us feel better. But the good news is that there are things you can do to feel better, and we want to help you feel better. I'll give you some tips, okay?

Strategy 1- Check what you do and what you feel: Can you notice if there are specific moments during the day when you feel worse (sadder and more discouraged)?

A first step is to IDENTIFY THESE MOMENTS AND VERIFY WHAT YOU'RE DOING IN THOSE TIMES WHEN YOU FEEL WORST. This will help you to realize that there are situations that make you worse and others that make you feel better doing certain things or tasks. These things or tasks can even be done when you are not very well, in order to help you improve your mood at that moment. In that case, I would like to make a deal with you: that every hour of the day you try to write on a sheet of paper or on a note on your cell phone what you did and your feeling at the time, or assigning a number to that feeling of 10 (I feel very good) to 1 (I feel very sad) to assess your mood. Do you think it would be possible to do this for a few days?

Strategy 2 - Discover (and rediscover) fun things: Usually when we are sad, we stop doing things, even the things we love to do. So, another very important thing at that moment is to DISCOVER OR REDiscover THINGS THAT AMUSE YOU. As hard as it is to think about fun now during this moment of so much uncertainty, don't forget that there will always be something that will make you feel a little better. So, I would like you to also make a list of all the fun things you used to do and enjoy. Can you do that?

Strategy 3 - Management of the environment: Aspects of the environment, that is, your room or your home, for example, can also make a difference at this time. So, always keep the environment well ventilated, when possible with sunlight and minimally organized. Staying most of the day in very dark environments or rooms may NOT be a good choice for you at this time. Therefore, how about we agree that you evaluate your environment for a day and, if possible, make small changes in habit or environment. It can be keeping the windows open and / or the environment lighter, organizing a cupboard, the wardrobe, changing the position / configuration furniture so that you are satisfied, etc. What do you think about this?

Very well. These were some initial suggestions for you to improve your mood. At another time, we will talk about this topic again and suggest more strategies like these for you. Next week I'll call you again and you tell me if it worked, okay? I also want you to remind you that you are doing very well. Is there anything else you want to ask me before hanging up?

**INTERVENTION WEEK 6**

Good afternoon Mr___, how are you? How was the last week? Were you able to put into practice our tips for improving feelings of sadness (ONLY IF THIS APPLIES)?

The purpose of our call today is to talk a little about how your diabetes is doing. (IF THE PATIENT DOES  HOME BLOOD GLUCOSE MONITORING) Have you written down your HBGM? Have you had hypoglycemia or hyperglycemia? (IF HE DOES NOT CONTROL, SKIP THE QUESTION).

IF HE'S PRESENTING HYPERGLYCEMIA: And did you notice any factors that may be causing these changes in your blood glucose? Have you been changing your diet? Are you managing to maintain a healthy diet? A balanced diet is essential for good control of your diabetes. Have you eaten at the usual time or are you having difficulties maintaining your routine?

IF HE HAS BEEN HYPOGLYCEMIA: Have you been feeling unwell when your glucose is low or don't you usually feel anything? Did you ever need to seek medical attention because of low blood glucose?

ABOUT MEDICATIONS: As for your medications, have you been able to use them correctly? Are you getting them from the pharmacy when you need them? Do you need a prescription? (if yes, ask for CPF, RG and email and forward to Janine)

If you had to tell me something that is still not good in the care of your diabetes, what would it be? (food, lack of routine, exercise, lack of tapes or medications ...). We will try to help more in this part in the coming weeks. In the meantime, let's remember to follow the tips I gave you in the last few weeks and try to put them into practice. Would you like me to send you the list on some specific subject we talked about?

**INTERVENTION WEEK 7**

Good afternoon Mr___, how are you? How are the things we agreed on in the previous links? The purpose of our call today is to talk a little about your sleep routine, especially if you are having trouble falling asleep, waking up a lot during the night, and as a result, you are not getting enough sleep. Would you like to hear some suggestions that we have that can improve this part or do you think this is not a problem and prefer to focus on other things? - If he replied that he doesn't think it's necessary, don't force it. Ask about diabetes, HBGM control, treatment goals and difficulties you are having. Something very general and similar to what was the first week. - If he answered yes, follow below.

Very well. Tell me briefly how your sleep routine has been going for the past few weeks? Yes, I understand! In some cases, nights can be very difficult as it is usually at this time that we tend to think about various things in our life, good things and bad things. Both increase our anxiety and as a consequence we may have difficulty falling asleep, waking up several times during the night. With that in mind, we want to help you sleep better. Having a good night's sleep will benefit your physical and mental health.

TIP 1 - Relaxing routine: Having a relaxing routine before bed can help you fall asleep more easily. So the idea is that you try, whenever possible, CREATE A QUIET MOMENT FOR YOU TO SLOW DOWN before going to bed. Some things you can do are: have a drink (tea, for example; but avoid stimulating drinks like green tea, coffee and chimarrão) or a hot bath to feel relaxed; turn off the lights, or if necessary leave as little light as possible in your room or home; make your bed very comfortable and cozy, and if possible only lie on it when you are really sleepy. These are some tips that can make you feel more relaxed before going to sleep and make it easier to fall asleep. How about testing these tips in the coming days? Then you tell me which one made you more relaxed before you went to sleep.

* If you already do this on a daily basis, keep doing it, as it is a great way to make you slow down, relax, and be more peaceful to sleep.

TIP 2 - Avoid electronic devices: This tip is in addition to the relaxing routine that I just told you. In addition to avoiding watching the news around bedtime, as we suggested in another call we made to you, another important thing is to AVOID THE USE OF ELECTRONIC DEVICES before going to sleep, and if that is not possible, at least you will reduce the device brightness that is stimulating and can make it difficult to fall asleep. But when you're already lying in bed, avoid this use anyway. This will help you to start sleeping more easily. Can we agree to try to do that in the next few days?

TIP 3 - Shifting the focus of thought: Our next strategy is for both when you go to sleep or for those times when you wake up at night and are unable to go back to sleep again. These are very favorable moments for you to be thinking about various things, and the more you listen to these thoughts, the more difficult it will be to return to sleep, even more if they are thoughts that cause you a lot of anxiety. That is why the main tip for this moment is to CHANGE THE FOCUS of the thought. You can do this by trying to focus on something else or by thinking about relaxing things. For example, put a song you like, in ambient sound; try to read a chapter of a book or magazine for a few minutes before falling asleep; exchanging anxious thoughts for restful, peaceful, relaxing thoughts; say prayers, if you have this practice. What do you think of that, could you try?

Great! These were some guidelines for you to improve your sleep. Again, if you think your sleep is still compromised, you can always call 188. Some professionals will be available to talk. Next week I'll call you again and you tell me if it worked, okay? Is there anything else you want to ask me before hanging up?

**INTERVENTION WEEK 8**

Good morning Mr. ____, how was your week? Did you put in practice the tips from last week? Have you noticed any improvement in your sleep pattern? If yes: Ok, good that there was an improvement, let's continue like this. If not: I see, a habit can take time to change. Ideally, you should insist on these tips I gave you, and over time your sleep may improve.

Today I came to give you some guidelines on exercise that combine a little with what we talked about in the last few weeks. Do you remember that we talked about eating habits? Did you know that exercise helps your body to prefer healthier foods and improve your meal routine? Another topic we talked about was mood and feelings of sadness, which can arise during this pandemic ... Did you know that exercise releases hormones that bring a sense of well-being and improve your mood? Even for the quality of sleep, which was our topic last week, regular exercise can help! Have you been able to exercise? Tell me a little more about it.

If he's doing it, POSITIVE REINFORCEMENT. If he is not succeeding, offer again the list of exercises to be sent by email.

Finally, I would like to remind you that it is important to avoid performing aerobic exercises (such as walking, running, cycling) at night, close to bedtime, as these increase brain oxygenation and this can make you very alert and make it difficult for you to fall asleep. So if you are going to exercise at night, choose the calmer ones or try to practice them earlier, at least 4 hours before the time you want to sleep. Always remember that, in addition to all the benefits we talked about today, physical exercise is essential for the treatment of diabetes, as it helps to maintain your blood glucose, in addition to controlling weight and blood pressure.

Do you have any other questions you would like to ask me? Now that you know about the many benefits of this habit, who knows, you may be excited to start / continue performing physical activities daily?

**INTERVENTION WEEK 9**

Good morning Mr._____, how was your week? How are the things we talked about last week going?

Today we're going to talk a little bit about blood pressure. Have you been able to measure your blood pressure during this pandemic period? If the answer is AFFIRMATIVE, ask how the blood pressure levels are. (write down and, if values change, talk to the team)

We have prepared some tips that can help to keep blood pressure at adequate levels. Would you like to hear them? (suggest writing down the tips).

TIP 1) Avoid adding salt to food. Try replacing it with other types of seasonings, which add flavor without interfering with pressure levels.

TIP 2) Try to reduce the intake of fats and fried foods, choose foods with low saturated fat (lean meat, fish and chicken breast). Invest in the intake of greens, legumes, oilseeds (nuts), seeds and grains.

TIP 3) If possible, try to maintain aerobic exercise (walking, cycling) daily, always remembering to take all precautions related to the coronavirus.

Okay, that's it for today. Is there anything else you would like to ask me?

**INTERVENTION WEEK 10**

Good afternoon Mr___, how are you? The purpose of our call today is to talk about stress, about that overload that we feel at times and that, over time, can be very harmful to our physical and mental health. Would you like to hear some suggestions that we have that can improve this part or do you think this is not a problem and prefer to focus on other things? - If he replied that he doesn't think it's necessary, don't force it. Ask about diabetes, HBGM control, treatment goals and difficulties you are having. Something very general and similar to what was the first week. - If he answered yes, follow below.

Very well. Now tell me: are you very stressed, overwhelmed? And what do you attribute all this stress to? Can you identify (the stressors) what makes you so stressed? And what do you feel when you are stressed, what changes in your physical and mental health? (let the patient speak for a few minutes, try not to interrupt initially).

I understand! We all experience stress, but when it is very intense and lasts for a long time, it can cause a lot of damage to our health, including making it difficult to control diabetes. So, today I would like to teach you some tips that can help you deal with stress, especially when we cannot change the stressor, that is, when we cannot change that / situation that causes us stress.

Strategy 1 - Helplessness and Escape: When you feel very overwhelmed / stressed, you can also feel exhaustion, that feeling that you have reached your limit. With that, you end up paralyzed in the face of stressful situations and problems, DEFERRING OR GIVING BACK OF LOOKING FOR SOLUTIONS. This sensation, when persistent, can generate losses and many negative feelings. So, the first tip for today is if you are having difficulty organizing and following your daily schedule, your routine and solving your tasks. For this situation, the tip is that you, at first, “take it easy on yourself”. In other words, avoid blaming yourself or criticizing yourself, after all, the moment is difficult and requires adaptation for everyone. In addition, I would like to agree with you that, during these moments, you remember our conversation today and try to understand / identify the reasons that made you not follow your planning / routine, and that you seek at least one alternative to modify / resolve this to the next few days. Shall we combine this?

Strategy 2 - Avoid conflicts: Some signs tell us when we are very stressed, and one of these signs is evident in the way we behave. For example, we fight and argue more with people or lose patience with them more often. So, the second tip for today is to AVOID DISCUSSIONS AND FIGHTS, as this tends to increase your stress, decrease the collaboration of other people and also harm interpersonal relationships. Remembering hurts, identifying culprits are other examples of attitudes that can make you feel angry and resentful. This will not help you in those moments and can lead to inaction and problem solving, as we commented in strategy 1. So, the second tip is that when you feel like this, pay attention to when those thoughts and attitudes appear. The idea is that you find a way to deal with this discomfort, always remembering that thoughts and feelings may not fully represent reality. One way can be using one of the relaxation strategies we talked about in another connection, remember? They can help you to reduce impulsivity and anxiety. What do you think, can you try to find a way to tolerate these discomforts and avoid conflicts?

Strategy 3 - Acceptance: There are stressful situations, that is, those that cause us stress, which we cannot change. In this case, the best thing to do is to ACCEPT THE SITUATION, try to give it another meaning and also regulate our emotions, negotiating with ourselves what is possible to do in the face of the situation, even if they are small things. This can help us to improve our perception of control of the situation. If you are living and feeling stressed due to one of these situations that cannot be changed, some tips are: (1) accept the situation realistically and without judgment, since accepting it is an important step to understand and act in what it really needs to be done (focus on the problem and not on the emotion!); (2) share with someone you trust how you feel, the search for social support is important to deal with stress and a way to make you externalize your feelings; (3) give another meaning to the situation, try to find some positive aspect or possible advantages / learnings that you have acquired from it. Did you write down all these tips? Can you try to do that? During our next calls, can you tell me if any of these tips worked for you, okay?

So, these are some stress management tips that you can use. Again, if you feel that you still need expert help, you can always call 188. Some professionals will be available to talk to you at any time, okay? You should already have that number written down, okay? Would you like to write it down again?

**INTERVENTION WEEK 11**

Good afternoon Mr___, how are you? How was your week? Have you been able to get your medications at the pharmacy and take them? Are you having any difficulties that I can help?

The purpose of our conversation today is to talk a little about the complications of diabetes. Has your doctor ever told you about having any complications from this disease? Tell me a little bit about what you know about it.

I'm going to explain a little bit to you about this and talk about things that can be done to prevent you from developing complications in the future. When diabetes is not well controlled, that is, when blood sugar is high for a long time and without proper treatment, it can end up causing injuries in some parts of the body. Blood vessels and nerves are the most affected when blood sugar remains so high. All of this can lead to a change in sensitivity, especially in the feet and legs, making you, for example, lose your slipper and not even notice (but it can also happen with your hands and arms). This can be very serious, as it is possible to develop wounds without your noticing, which can end up infected and take a long time to heal. Other things that inadequate glycemic control can cause are changes in vision, changes in kidney function and increased risk of cardiovascular diseases, such as heart attack, stroke and peripheral vascular disease.

To reduce the chance of these complications occurring, it is essential to use the recommended treatments correctly and take care of eating habits to avoid blood sugar spikes during the day! It is very important that you also maintain a physical exercise routine, according to your ability to do this without feeling pain or discomfort, in order to better control diabetes and reduce the chance of presenting all these things that I told you. In addition to taking care of these aspects at home, it is very important that you attend medical appointments regularly so that your doctor is aware of the appearance of any complications and initiates appropriate treatment in case of any problem. Also, be aware of the sensitivity of your feet: notice if you start to feel pain, heaviness, tingling or lose sensation; also take care of the quality of your vision: notice if you start to see less or have blurred vision. Always tell your doctor and talk about any changes you notice at home. The sooner you talk to him, the sooner it will be possible to start the proper treatment avoiding more serious complications.

That's what I wanted to talk to you about today. Do you have any questions? Anything else I can help you with?

**INTERVENTION WEEK 12**

Good afternoon Mr___, how are you? Have you been able to do the things we agreed on?

The purpose of our call today is to talk about the relationship between thought, emotion and food; about hunger, desire to eat. Would you like to hear some suggestions that we have that can improve this part or do you think this is not a problem and prefer to focus on other things? - If he replied that he doesn't think it's necessary, don't force it. Ask about diabetes, HBGM control, treatment goals and difficulties you are having. Something very general and similar to what was the first week. - If he answered yes, follow below.

Very well. Now I would like to know what your relationship with food is like, that is, do you think you overeat often? Do you think you eat more or less than you should? (let the patient speak for a few minutes, try not to interrupt initially).

Yes, I understand! The way we relate to food is closely associated with our thoughts and emotions. Perhaps you have already felt very happy or very sad and then decided to eat much more than usual; or the other way around, you’ve lost the will to eat and didn’t eat or ate much less than usual. So, today I would like to present you with some tips so that you can first understand how this relationship (thought / emotion / behavior of eating) is for you.

Strategy 1 - Identifying sabotaging thoughts: Do you remember that in our other phone calls I explained to you that there is always a thought that precedes our way of feeling things and the way we behave? So, eating behavior is no different. As much as you have the feeling of eating automatically, eating is not an automatic action. This means that you can learn to have more control over your eating decisions / behaviors. All the thoughts that make you act inappropriately are what we call SABOTAGING THOUGHTS. For example: "it is very difficult to follow the diet", "I can't resist this delicious food", "I know I shouldn't eat this, but I want it so much". So, today's tip is that you try to listen to your thoughts during the week before eating any type of food. At first it can be more complicated to identify what that thought is, but as you exercise this tip, the easier it will be to hear and identify those thoughts. Are you up for trying to do that?

Strategy 2 - Identifying stimuli: There are different stimuli that cause people to eat and that also lead us to have sabotaging thoughts before eating. These stimuli can be: environmental (sight and smell of food); biological (hunger, thirst, uncontrollable desire to eat); mental (thinking about food, reading a cooking recipe, remembering a food you ate and liked,); emotional (negative feelings such as anger, anxiety, frustration, sadness, which lead to eating to seek comfort or distraction. Positive feelings are also emotional stimuli!); and social (situations and / or people that encourage us to eat). With that, the second tip is to try to IDENTIFY WHICH STIMULUS make you have sabotaging thoughts and inappropriate behavior in relation to food. This identification is important, as you can reduce your exposure to them or change the way you deal with them. You can also write down the stimuli and thoughts you identify. Remember that the first step in changing your behavior is to understand how it occurs.

Strategy 3 - Differentiation between hunger x desire to eat x uncontrollable desire: Another important aspect is knowing how to differentiate the feeling of hunger with the other sensations that are similar to it. HUNGER is when you experience a feeling of emptiness in your stomach that is often accompanied by noise. THE DESIRE TO EAT is when you want to eat something because you are being influenced by other stimuli (such as environmental) even if you have just eaten. In UNCONTROLLABLE DESIRE you feel an urge to eat a specific type of food, accompanied by tension or an unpleasant feeling in your mouth, throat or body. Knowing this differentiation can help you make the right food decisions. So, the tip is: before eating outside the fixed meal times needed during the day, try to identify if what you are feeling is really hungry, if you want to eat only or if it is an uncontrollable desire. Can you try that?

These are some initial tips on the relationship between thinking, emotion and eating / eating behavior. At another time, we will talk about this topic again and suggest more strategies for you. Any questions?

**INTERVENTION WEEK 13**

Good morning, how are you? How was the week? Mr ___, today I would like to talk a little about your perceptions about the pandemic and diabetes. Do you feel that the pandemic has affected your diabetes care? (ask to explain).

What are the main difficulties you have experienced during the pandemic? How do you think you could improve this? (For researchers, write down and tell Janine what the main difficulties are for us to think about any intervention on what is most prevalent!)

Have you been able to obtain the medications? Are you taking them at the right time?

Do you control your HBGM? Can you give me the values you've been presenting?

How's your blood pressure going? Have you checked at home? If so, what values are you presenting?

Today our conversation was shorter. We are in the final stretch of our research, soon our phone calls will end. Is there anything that you think is important to discuss before we end the calls?

**INTERVENTION WEEK 14**

Good afternoon Mr___, how are you? How were the last few weeks?

The purpose of our call today is to talk about eating behavior again; mainly about organizing the environment and eating habits. Would you like to hear some suggestions that we have that can improve this part or do you think this is not a problem and prefer to focus on other things? - If he replied that he doesn't think it's necessary, don't force it. Ask about diabetes, HBGM control, treatment goals and difficulties you are having. Something very general and similar to what was the first week. - If he answered yes, follow below.

Okay. Now I’d like to know what your food habits are like? Do you have a habit of eating standing up? How long do you take to eat? And how is the food organization in your home?

Some habits related to our behavior during meals and even the way we organize food in our home can hinder us, especially if we have difficulty following and maintaining a healthy or specific diet, for example. Starting with small changes in habits and in the environment can be a good strategy if you are having difficulties in achieving any of these goals. So, the three tips today are:

TIP 1 - Sit down to eat: Sitting down to eat is a very important behavior, especially to be aware of the amount of food we put in the mouth. Most of the time, when we eat standing up, we eat on impulse instead of choosing foods that are part of your planned food / meal; not to mention speed and distraction. Paying attention to what you are eating can help you not let sabotaging thoughts like “I'm still hungry”, “I want more”, convince you to eat more than you should. SITTING, chewing and tasting food more calmly will also give you a degree of satisfaction. Therefore, the first tip is that if you have this habit, try to sit down to eat from today, even if it is during that afternoon or evening snack. What do you think?

TIP 2 - Eat slowly and consciously: Complementarily to the previous strategy / tip, the second tip is: eat slowly and consciously. This alone has several benefits: when you eat SLOW, your brain has time to register that you are satisfied; or even when you notice and ENJOY EACH PORTION OF FOOD, you feel more satisfied after eating. Research indicates that there is an interval of up to 20 minutes between the moment your stomach is full of food and the recognition of this by the brain; moreover, people who eat more slowly tend to eat less. So, if your problem is overeating, how about exercising this strategy?

TIP 3 - Organize the environment: The way food is displayed in your kitchen or the size of the utensils you use can make a difference when it comes to controlling the urge to eat something tempting or even the amount of food you eat. it suits. For example, some people overeat when using cutlery and large plates. If so, how about trying to REORGANIZE the kitchen utensils in your home, so that small utensils can be easily accessible? So you can use smaller cutlery and plates more easily in your daily routine. Another tip is in relation to FOOD ORGANIZATION. If foods that are tempting to you are visibly displayed in kitchen cabinets, an alternative is to place them on the back of a cupboard / shelf or in a tall cupboard / shelf. The idea is to reduce the visual stimuli that can cause you to overdo and eat more than you should and at inappropriate times. You can also ask someone else in your home to do this reorganization. And when possible, avoid buying these foods that are considered tempting, especially in large quantities / portions. Shall we try to do that?

These are today's tips, I hope they can help you better organize your environment and habits at mealtimes. Is there anything else you would like to ask?

**INTERVENTION WEEK 15**

Hello Mr ____ How are you? Today is one of our last call, and I wanted to know how you spent the week.

{if he does HBGM control} Were you able to do your glycemic control this week? If you check your blood sugar, would you like to give me the HBGM values?

If he had hypoglycemia: Did you have any symptoms that made you realize you were hypoglycemic? What did you drink or eat to manage this? And then it was okay? Did you need medical attention?

If he had hyperglycemia: Can you identify why the levels of glucose were so high these days? If he can't: let's think together, did you eat anything different this week? Do you think you overeat during a meal or ate out of hours? Did you forget the medications? Did you change your habit of exercising, did you stop practicing? Well, now that we can identify the cause, we can correct this behavior. What do you think you can do? Can we do this agreement then? (if the patient has not responded, help him think of a strategy).

Are you getting diabetes medications? What about the other medications? Are you managing to take all your medicines at the right time?

We are almost at the end of our study, next week will be our last call to give guidance and talk a little more about what your experience was during the pandemic. Do you think our calls helped you to take better care of your diabetes and make you feel more relaxed during quarantine?

Very well. Is there anything else you would like to ask or that I can help you with?

**INTERVENTION WEEK 16**

Good morning Mr_____. Today is our last call about our diabetes research during the pandemic. We had the opportunity to talk about a lot of things during those months of conversation. Do you think our calls helped in any way? Is there anything in particular that you would like to remember or talk about a little more today that is our last call?

RESUME PREVIOUS INTERVENTIONS, as the patient chooses or finds necessary:

Exercise: FIRST TIP: have a place available at your home to perform your exercise practice at any time. Often the lack of a place available prevents us from practicing because we have to move furniture around and create an environment that allows us to move around the house every time we decide to exercise. The space doesn't have to be big, just enough for you to move around without hitting furniture. If you do not have space at home, you can perform exercises such as walking outdoors, but choose to go out at times when the circulation of people is not much, and try to keep a distance of two meters from people. Preferably, use a mask.

SECOND TIP: Try to create a routine for your body to get used to the fact that every day at that time you will be exercising. If you are having trouble keeping a schedule, or starting the practice, try doing it in a more timely manner, without having to stop doing what you are doing to go and practice. For example: practice in the intervals of a program you are watching, or practice while watching a series or movie, so it can be easier to maintain a routine.

THIRD TIP: If you haven't started exercising yet and find it a little difficult, start like this: in the first week, do 5 minutes of exercise every day. In the second week, increase to 10min. The following week, 15 min, and so on, until you reach at least 30 min of daily practice. It is important to remember that you can perform aerobic exercises, such as walking, running, skipping rope, or anaerobic exercises, such as those for weight training.

A VERY IMPORTANT detail is that you should always check your blood glucose before starting activity and, if possible, intensify monitoring during the day, especially if you have done an unusual exercise. It is also important to keep some fast-acting carbohydrates nearby to correct any hypoglycemia. And don't forget about hydration, also an important attitude to preserve immunity.

HEALTHY EATING:

FIRST TIP: Eat fresh, natural foods every day. Try to maintain a regular consumption of fruits, vegetables and whole grain products daily. The recommended thing is to try to eat 4 servings of fruits per day, 5 servings of vegetables, 1 to 2 servings of grains and meat at least 2 to 3 times a week. In addition to being eaten fresh, fruits and vegetables can be frozen. Another idea is to use vegetables to cook large quantities of soups and other dishes and then freeze the ready-made meal, only reheating when you eat it. This will make it last longer and provide meal options for a few days.

SECOND TIP: When it is difficult to get natural products, opt for healthy dry or canned alternatives. Canned beans and chickpeas, which provide various nutrients, can be stored for months and can be included in meals in a variety of ways. Canned oily fish, such as sardines, mackerel and salmon, are rich in protein, omega 3 fatty acids and a variety of vitamins and minerals. Dry products such as beans, legumes and grains such as lentils, peas, rice, or quinoa are also nutritious, long-lasting and tasty options.

THIRD TIP: Create a stock of healthy snacks. Instead of keeping sweets or snacks at home, choose to keep a stock of healthier options, such as nuts, yogurt (without sugar), chopped or dried fruits or hard-boiled eggs. These foods are nutritious and will keep you full for longer. Try to avoid sugary drinks and try to drink lots of water throughout the day.

MENTAL HEALTH: (Strategy 1 - Managing the environment): Do you usually watch news on television or other media? The truth is that the news is today the main enemy of our “mental tranquility”. The news keeps reminding our brain of the “threat” that we are living with the coronavirus. This activates a series of responses in our body that make us feel anxious. The first step in changing this is to CONTROL OUR NEWS CONSUMPTION. Some things are fundamental in this part and you should write them down. First, let's agree that you will watch news for a maximum of 2 hours a day (from morning to night). Second, let's agree that you will not watch news for at least 2 hours before going to sleep (that is, if you usually sleep at 10 pm, ideally you should stop watching news after 8 pm) - this will prevent negative thoughts in your head at bedtime, causing discomfort and disrupting sleep.

(Strategy 2 - Distraction) Reducing news consumption is very important, but that alone will not be enough. Can we agree that, thirdly, whenever thoughts of concern and fear about the coronavirus arise in your head, you will automatically shift your focus from thinking to something good? Every time you start to get anxious, you should close your eyes, take a deep breath, and make a mental list of 3 things you like most, that make you most happy.

(Strategy 3 - Social relations): What will help you most is keeping in touch with your friends. I know it is very difficult now, in the quarantine season, but talking to someone you like every day will help to alleviate this anguish that you may be feeling. Can we agree that you will try to talk to at least one person every day (it can be over the phone, or by text message on your cell phone or computer)? It has to be a person that you don't see daily.

Before we finish our conversation, there are some important things that we need to agree on. We haven't had a blood test in a long time to see how your diabetes is doing. As our research aims to help in this part, we organize a safe way for you to be able to go to the hospital - by appointment - and take the exam without having to stand in lines and WITHOUT CONTACTING ANY OTHER PATIENT (reinforce that it is safe!) . For this, we schedule a blood test that will be done in a special room, which is next to the Hospital de Clínicas. One of our researchers will be waiting for you at the entrance to this site to measure your blood pressure, deliver your exam order and show you where the sample collection is. Can you get there? Take a pen and I will give you the time and the name of the place: (Clinical Research Center (CPC) of Hospital de Clínicas de Porto Alegre - day ___ CHECK TABLE!). Don't forget this date, we were able to book especially for you to be able to take exams safely!

Very well. In addition to this exam, in the coming weeks we will make a final call to apply those questionnaires we talked about at the beginning of the survey. It will be a longer call, but it will be the last. Okay? Is there a time that you prefer that I call to apply the questionnaires?

Thank you very much for your patience during all this research time. It was a pleasure to be able to talk and help from a distance! Have a good week.

*These protocols were published as supplementary material at doi: 10.1007/s00592-021-01690-1, which described the intervention performed among patients with type 2 diabetes. In this manuscript, the impact of the intervention among patients with type 1 diabetes is described.
